# Outline of the principles of management of cardiac tumours

**DOI:** 10.1186/1749-8090-10-S1-A166

**Published:** 2015-12-16

**Authors:** Philemon Gukop, Oswaldo Valentia, Goppal Soppa, Aziz Momin, Justin Nowell, Robin Kanagasabay Mazin Sarsam, V Chadrasekaran, Marjan Jahangiri

**Affiliations:** 1 Department of Cardiothoracic Surgery, St George's University Hospital NHS Foundation Trust, London, SW17 0QT, UK

## Background/Introduction

Primary cardiac tumours are important sources of morbidity and mortality. Most are benign, the malignant types being rapidly fatal.

Their clinical features are often non-specific. They could cause thromboembolic events, cardiac failure, arrhythmias and death. Only few case reports and case series exist on the subject. There is a need to outline a robust management protocol for this important disease. We describe our 20 years' experience of this pathology and outline the management principles.

## Aims/Objectives

To outline the principles of management of cardiac tumours

## Method

Retrospective data analysis on 78 patients who had surgery for cardiac tumours over 20 years period in a single centre, with prospective follow-up. The histological types and correlation of clinical and pathological diagnosis were determined. Data was analysed to determine the sensitivity and specificity of echocardiogram for diagnosis

## Results

78 patients, female (67%) mean age (61) myxoma (78%), malignant (8%). Atrial fibrillation (26%), stroke (23%),NYHA 2-3 (65%). Kaplan Meier curve showed malignant tumour are rapidly fatal within 1 year of diagnosis and myxoma does not significantly affect survival (p = 0.043). Echocardiogram has sensitivity (0.93) and specificity (0.95), accuracy (0.94) for detection of myxoma but very poor for diagnosis of malignant cardiac tumours, p = 0.0001.

## Discussion/Conclusion

More attention should be given to the management of cardiac tumours especially the malignant type with high fatality. Echocardiogram is not sensitive for malignant tumours.

The first principle in treatment of cardiac tumours is prevention and treatment of arrhythmias and cardiac failure.

The second principle is anticoagulation against thromboembolism events.

The third principle is prompt exclusion of malignancy by MRI scan (sensitive) and histology. Malignant lesions will require a staging CT scan to assess resect-ability.

The fourth principle is frozen section guided complete excision of resect-able lesions and oncological advice for malignant disease.

